# Supplementary material for: Whole body periodic acceleration in normal and reduced mucociliary clearance of conscious sheep
Source: PLoS One. 2019 Nov 7;14(11):e0224764. doi: 10.1371/journal.pone.0224764 (PMC6837306; doi:10.1371/journal.pone.0224764)
Supplement: Attachment — Submitted filename: Response to Reviewers.docx [file pone.0224764.s002.docx]

**Response to reviewers**

**Reviewer #1:**

We thank the reviewer for his/her laudatory comments on the scientific merit, content and writing of the manuscript and the acknowledgement of clinical utility.

A few issues, however, need to be addressed:

- Specific statistical test used for each figure should be included in the figure legend.

For an unknown reason, these were not uploaded to the manuscript submitted, they are now included in the legend.

- Tracheal mucus velocity (TMV) was used in the current study as an index of MCC. How changes in TMV correlate with whole-lung clearance? Please explain.

TMV reflects changes in whole lung clearance measured with radioactive labeled human serum albumin.(1, 2)

- Fig. 1 shows TMV modulation by WPBA-mediated NO production over a 2 h period post-WBPA. Did the authors try to get data for 2 other additional hours (time point 4 h)? Since there is a decrease in TMV values from 1 to 2 h, I am wondering if values would continue decreasing over time which might indicate a lack of NO production due to substrate depletion (decreasing NO bioavailability).

Low arginine bioavailability plays a pivotal role in the pathogenesis of a growing number of varied diseases, including sickle cell disease, thalassemia, malaria, acute asthma, cystic fibrosis, pulmonary hypertension, cardiovascular disease, certain cancers, and trauma, among others. Catabolism of arginine by arginase enzymes is the most common cause of an acquired arginine deficiency syndrome, frequently contributing to endothelial dysfunction and/or T‐cell dysfunction, depending on the clinical scenario and disease state. (3) None of these factors would be expected in healthy sheep. However, the duration of effects on TMV requires further study and was not addressed.

- Experimental plan shown in Figs 3&4 was prolonged for 4 hours. Were there any signs of irritation and/or drying of the airway from the beginning to the end of the experiments?

There were no obvious signs of airway irritation (coughing or hypersecretion) observed throughout the study. In order to reduce the effects of airway desiccation the animal was placed on room temperature humidify air between measurements

- Authors conclude (page 13, line 282) that NO released into the circulation from eNOS via pulsatile flow as a consequence of WBPA accesses the airway epithelium and improves MCC; this is based on pharmacological inhibition with L-NAME (a broad NO inhibitor) rather than direct eNO measurements. Is there any evidence for iNOS or nNOS involvement under these experimental conditions?

As mentioned by reviewer L-NAME also inhibits nNOS which is constitutively present in the mucous cells of the epithelium. (4) In rats. eNOS is phosphorylated at Ser-1177. eNOS phosphorylation (Ser-1177) was increased to 261% of control by 1 h of WBPA (360 cpm). Compared to controls, a single 1-h exposure to WBPA (360 cpm) increased the protein level of eNOS in the heart by 393 ± 85% and 461 ± 78% at 4 and 24 h, respectively. The increases of nNOS were 167 ± 9% and 189 ± 36% at 4 and 24 h, respectively. Consistent with these results, mRNA levels of eNOS and nNOS were also significantly increased by WBPA at a frequency of 600 cpm which caused a more sustained increase of eNOS at 4 and 24 h, 392 ± 46% and 534 ± 57% but significantly less induction of nNOS levels (135 ± 19% at 4 h and no change at 24 h compared to WBPA at a frequency of 360 cpm. Inducible nitric oxide synthase (iNOS) was not detected in control or with WBPA at either frequency. Moreover, we did not measure nNOS in airway epithelium with administration of WBPA.

**Reviewer #2:**

We thank the reviewer for their acknowledgement on our study design and clarity of our findings.

The significance of this work is the fact that other nitric oxide (NO) donors drugs increase ciliary beat frequency of nasal epithelium without increasing mucociliary clearance but WPA clearly increases MCC. This is possibly due to the fact that the NO release is more significant as WPA increases endothelial NO throughout the body. Mechanistically, they also clearly show that this is a NO dependent phenomenon and L-NAME blunts this effect. Overall, this is a well-designed study that clearly shows benefit from a simple non-invasive technique to temporarily increase NO levels and NO released systemically impacts the airways.

Concerns relate to the duration of WPA and the lack of discussion regarding shorter/longer duration. Can you quantify systemic increase in NO levels? Adding discussion regarding why this technique outperforms other NO donor drugs may also help.

The action of WBPA releasing NO into circulation as measured by descent of the dicrotic notch of finger pulse of humans begins within 15 to 25 seconds after beginning WBPA. (5) The duration of NO action is complicated because NO rapidly binds to circulating proteins and is slowly released to the body in a heterogeneous manner. *The effects of 1 hr of WBPA on eNOS expression and phosphorylation have been previously published in rats and mice (6, 7)*

We could not find any references to NO donor drugs on MCC in airways and cannot respond to question as to difference in performance. Runer and Lindberg showed increases in ciliary beating frequencies with nitroprusside on human nasal mucosa. (8)

We could not quantify acute systemic increases of NO as reflected by others using serum nitrite measurements because of inconsistent results present in our attempts to make this measurement*.*

Also, wouldn't continual administration of HNE represent a situation similar to that seen in inflammatory lung diseases?

This is an interesting observation and a reasonable possibility; however we have not attempted to do so, since our sheep model is not a terminal model. Prolonged administration of HNE, may in fact produce a very severe inflammatory lung disease from which there is no survival

Reference

1.	Hirsh AJ, Zhang J, Zamurs A, Fleegle J, Thelin WR, Caldwell RA, et al. Pharmacological properties of N-(3,5-diamino-6-chloropyrazine-2-carbonyl)-N'-4-[4-(2,3-dihydroxypropoxy)phenyl] butyl-guanidine methanesulfonate (552-02), a novel epithelial sodium channel blocker with potential clinical efficacy for cystic fibrosis lung disease. The Journal of pharmacology and experimental therapeutics. 2008;325(1):77-88. doi: 10.1124/jpet.107.130443. PubMed PMID: 18218832.

2.	Sabater JR, Mao YM, Shaffer C, James MK, O'Riordan TG, Abraham WM. Aerosolization of P2Y(2)-receptor agonists enhances mucociliary clearance in sheep. J Appl Physiol (1985). 1999;87(6):2191-6. doi: 10.1152/jappl.1999.87.6.2191. PubMed PMID: 10601167.

3.	Morris CR, Hamilton-Reeves J, Martindale RG, Sarav M, Ochoa Gautier JB. Acquired Amino Acid Deficiencies: A Focus on Arginine and Glutamine. Nutr Clin Pract. 2017;32(1_suppl):30S-47S. doi: 10.1177/0884533617691250. PubMed PMID: 28388380.

4.	Donaldson SH, Bennett WD, Zeman KL, Knowles MR, Tarran R, Boucher RC. Mucus clearance and lung function in cystic fibrosis with hypertonic saline. The New England journal of medicine. 2006;354(3):241-50. doi: 10.1056/NEJMoa043891. PubMed PMID: 16421365.

5.	Sackner MA, Gummels E, Adams JA. Nitric oxide is released into circulation with whole-body, periodic acceleration. Chest. 2005;127(1):30-9. doi: 10.1378/chest.127.1.30. PubMed PMID: 15653959.

6.	Wu H, Jin Y, Arias J, Bassuk J, Uryash A, Kurlansky P, et al. In vivo upregulation of nitric oxide synthases in healthy rats. Nitric Oxide. 2009;21(1):63-8. doi: 10.1016/j.niox.2009.05.004. PubMed PMID: 19481168; PubMed Central PMCID: PMCPMC3135669.

7.	Uryash A, Bassuk J, Kurlansky P, Altamirano F, Lopez JR, Adams JA. Antioxidant Properties of Whole Body Periodic Acceleration (pGz). PLoS One. 2015;10(7):e0131392. doi: 10.1371/journal.pone.0131392. PubMed PMID: 26133377; PubMed Central PMCID: PMCPMC4489838.

8.	Runer T, Lindberg S. Effects of nitric oxide on blood flow and mucociliary activity in the human nose. Ann Otol Rhinol Laryngol. 1998;107(1):40-6. doi: 10.1177/000348949810700108. PubMed PMID: 9439387.
